# Can Clays in Livestock Feed Promote Antibiotic Resistance and Virulence in Pathogenic Bacteria?

**DOI:** 10.3390/antibiotics4030299

**Published:** 2015-07-16

**Authors:** Alexandro Rodríguez-Rojas, Jerónimo Rodríguez-Beltrán, José Ramón Valverde, Jesús Blázquez

**Affiliations:** 1 Institut für Biologie, Freie Universität Berlin, Königin-Luise-Str. 1-3-14195 Berlin, Germany; 2 Instituto de Biomedicina de Sevilla (IBIS), Campus HU Virgen del Rocío. Avda. Manuel Siurot S/N, 41013 Seville, Spain; E-Mail: jrodriguez-ibis@us.es; 3 Centro Nacional de Biotecnología (CNB), Consejo Superior de Investigaciones Científicas (CSIC). Calle Darwin 3, 28049 Madrid, Spain; E-Mail: jrvalverde@cnb.csic.es

**Keywords:** antimicrobial, antibiotic resistance, horizontal gene transfer, virulence factor, clay, sepiolite

## Abstract

The use of antibiotics in animal husbandry has long been associated with the appearance of antibiotic resistance and virulence factor determinants. Nonetheless, the number of cases of human infection involving resistant or virulent microorganisms that originate in farms is increasing. While many antibiotics have been banned as dietary supplements in some countries, other additives thought to be innocuous in terms of the development and spread of antibiotic resistance are used as growth promoters. In fact, several clay materials are routinely added to animal feed with the aim of improving growth and animal product quality. However, recent findings suggest that sepiolite, a clay additive, mediates the direct transfer of plasmids between different bacterial species. We therefore hypothesize that clays present in animal feed facilitate the horizontal transfer of resistance determinants in the digestive tract of farm animals.

## 1. Introduction

The exposure of bacteria to inhibitory concentrations of antibiotics results in the selection of resistant sub-populations. In addition, sub-inhibitory levels of many antibiotics raise mutation and recombination rates in bacteria, increasing the probability of resistance acquisition, even to unrelated antibiotics [1,2]. Antimicrobial agents also promote the acquisition of DNA sequences from other organisms via horizontal gene transfer (HGT) [1,3]. HGT plays a major role in pathogen evolution by allowing bacteria to evade the immune response and distributing genes that increase virulence or provide increased resistance to antibiotics [4]. Indeed, there is now much concern surrounding the huge quantity of antimicrobial agents used as livestock growth promoters and the possibility that they select for resistance determinants, which can spread by different routes to human pathogens compromising antibiotic efficacy [5,6]. Furthermore, antibiotic resistance genes conferring resistance to several antimicrobials have been used for the construction of some genetically modified plants which are used as animal feed [7]. The possibility of gene transference via HGT to the microbiota and subsequently to the community must therefore be examined [8].

Animal feed also contains other supplements such as certain clays. Sepiolite, for example, was authorized for use by the European Union in 1990 and registered as technological additive E-562. The purpose of these minerals is to make digestion more efficient. These additives are believed to improve growth and animal product quality, and are widely used in feed for broiler chickens [9] and pigs [10]. Such practices are considered innocuous. Clays, including sepiolite, have also been proposed for use in a large number of pharmaceutical applications [11,12]. Interestingly, due to their excellent adsorption capacities, clays have been proposed as carriers for antibiotics among other compounds [11]. Furthermore, certain clays present some antibacterial effects, due to the presence of bactericidal metallic cations (mostly Fe^2+^ or Cu^2+^) [13] either of natural origin [14], or adsorbed artificially [15]. Scientific literature states that they fulfill all safety, stability, and chemical inertia requirements, and their presence in tablets used in human medicine has been established as safe and without side effects [11].

A relationship between the presence of sand or clay minerals and bacterial genetic exchange has been reported, and the possibility that mineral-mediated DNA transfer between different bacterial species plays a role in the evolution of antibiotic resistance has received some attention [16]. Transformation of bacteria by foreign DNA in the presence of friction forces and clay materials is known as the Yoshida effect [17]. This transformation relies on the ability of mineral nanofibers (or nanoneedles), such as those formed by sepiolite, to adsorb DNA and form a chestnut burr-shaped complex when a sliding friction force [18] or even vibrations [16] are applied. These complexes can penetrate the bacterial cell wall and membrane, releasing DNA into the cytoplasm [19]. This effect was observed in many bacterial species (Table 1).

**Table 1:** Bacterial species in which clay-mediated transformation has been examined.

Bacterial Species	References
*Pseudomonas aeruginosa*	[20]
*Salmonella enterica* Serovar Typhimurium	[20]
*Mycobacterium smegmatis*	[20]
*Pseudomonas putida*	[21]
*Escherichia coli* (several strains)	[21,22,23]
*Yersinia enterocolitica*	[23]
*Acinetobacter baumannii*	[23]
*Bacillus subtilis*	[16]
*Pseudomonas elodea*	[16]

Here we propose that “recipient” bacteria can be transformed by DNA fragments or plasmids from “donor” bacteria adsorbed onto mechanically-penetrating mineral nanofibers. Laboratory work has already provided proof of concept of this idea; the exclusive combination of friction forces and sepiolite allows plasmids to be easily transferred between bacterial species, including strains of *Escherichia coli*, *Salmonella enterica* (serovar Typhimurium), *Pseudomonas aeruginosa* and *Mycobacterium smegmatis* [24]. These experiments were designed in such a way that different antibiotic-resistance markers could be used to counterselect donor bacteria. The results clearly demonstrated that DNA transfer between bacteria was mediated by sepiolite.

## 2. The Hypothesis

**Figure 1:**
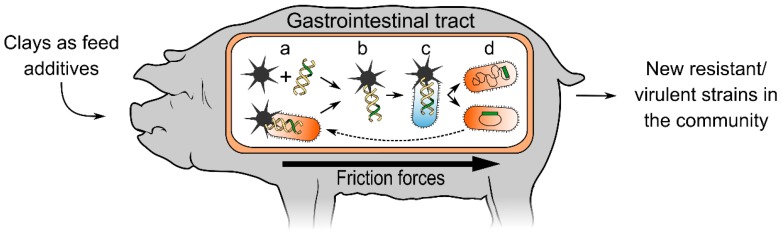
Clay-mediated DNA transfer in livestock. Clay additives in animal feed form complexes due to friction forces that may promote the release of DNA from bacteria. This free DNA can then be adsorbed by clay (a). The resulting DNA-clay complexes (b) penetrate other microbes, internally delivering the carried DNA (c). This may result in the acquisition of new virulence genes and/or antibiotic resistance determinants (d). Both commensal and pathogenic bacteria may act as recipients or donors and acquire traits that compromise the treatment of infections.

The combination of clays present in animal feed, the presence of both pathogenic and commensal bacteria in the animal gut, the mechanical friction provided by peristalsis in the rumen and intestines (or the strong abrasive action of the gizzard in poultry), and the routine administration of antibiotics to livestock (providing the selective force required for the fixation of antibiotic resistance), led us to hypothesize that clay-mediated DNA transfer among bacteria may occur in farm animals. This phenomenon could provide an additional HGT mechanism able to disseminate antibiotic resistance and virulence genes across species (Figure 1).

The presence of antibiotics would not only provide the selective pressure to fix transformed cells in their populations, but is also an additional source of DNA via cell lysis. Plasmid DNA, which does not need to integrate into a recipient bacterium’s chromosome, might be efficiently introduced by clay-mediated transfer. Mobile elements such as transposons or integrons also become active in the presence of antibiotics [25] and could be transferred with the help of clays. The fact that antibiotics increase rates of exogenous DNA recombination (*i.e.*, the frequency of integration of foreign DNA into the bacterial chromosome) [4,26] might indeed improve the likelihood of transformation.

Clay-mediated transfer of DNA have been experimentally demonstrated *in vitro* and the parameters necessary for its occurrence are relatively well characterized [17,22,27]. Despite the obvious differences with the environment found in the laboratory, the key conditions required for clay-mediated transference could be found in the gastrointestinal tract of farm animals. For example, the optimum temperature for DNA adsorption by clay materials is 37 °C [27], which is within the body temperature range of cattle. The friction forces required for transformation driven by clay-DNA complexes are common in the gastrointestinal tract of livestock. *In vitro*, an optimum transformation pressure of 3.9 kPa has been recorded [22]; such a pressure could easily be achieved in the gastrointestinal tract of most farm animals (Table 2).

**Table 2:** Pressure values exerted in different parts of the digestive tract of several livestock species. The optimal pressure for the clay-mediated transformation of bacteria by plasmid DNA is reported to be 3.9 kPa [22]. The numbers shown have been converted to the international system unit for pressure.

Average Pressure of Digestive Tract (kPa)	Species	Digestive Tract Section	References
2.7	humans	jejunum	[28]
2.7	humans	pylorus	[29]
10.7	humans	oesophagus	[30]
37.3	geese	gizzard	[31]
3.1	turkeys	muscular stomach	[32]
0.5–39.9	chickens	gizzard	[33]
3.9	chickens	muscular stomach	[32]
4.7	pigs	stomach	[34]

The gut environment is generally characterized by a low pH and an elevated osmolarity. These circumstances could, in principle, hamper DNA transfer, but clay-mediated transformation has been shown to be robust to such conditions as clay materials can adsorb DNA over a wide range of pH. Since DNA stability is compromised at highly acidic conditions, clay-adsorbed DNA is unable to transform bacterial cells at pH lower than 3 [27]. However, pH values found in the stomach of mammals and the gizzard of poultry are high enough (4.4 in pigs [35] and 3.5 in broiler chickens [36]) to allow DNA adsorption and transformation [27]. Furthermore, the pH in the intestine of livestock rises up to ~6 [35,36], a closer value to the adsorption and transformation optimum found *in vitro* [27]. Regarding salinity, clay-mediated transformation has been reported to occur at a range of 0–0.3 M of NaCl without a significant loss of efficiency *in vitro* [17]. The osmolarity in the gut, based on a complex mixture of salts, has been estimated to be equivalent to 0.3 M NaCl [37], which is, again, within the limits for optimal transformation *in vitro*. Taken together, the confluence of *in vitro* and *in vivo* data suggests that gastrointestinal tract is a plausible scenario for clay-mediated DNA transfer.

One might argue that if free DNA fragments were present in the digestive tract of animals, they would be rapidly degraded. On the other hand, it has been shown that clay-adsorbed DNA is very resistant to degradation in the gut and soil [16]. Additionally, it has been reported that DNA adsorption by clays provides protection against nucleases [27,38]. However, this protection seems to be only partial: it has been shown that clay-adsorbed plasmidic DNA treated with DNase I displayed a restricted availability for transformation, suggesting that DNA molecules were not intact. Yet, even partially degraded DNA could still serve as a substrate for integration into the recipient chromosome by recombination [38]. This protected DNA therefore provides a reservoir of genetic material that could benefit naturally-occurring competent bacteria in the digestive tract and allow clay-mediated transformation.

Previously discussed arguments and laboratory results obtained to date, provide sufficient evidence to recommend examining clay-mediated DNA transfer *in vivo*. Thus, we propose that the role of sepiolite and other clays as promoters of antibiotic resistance and virulence trait transfer in farm animals should be investigated. To what extent the proposed mechanism of clay-mediated HGT could operate *in vivo* is difficult to answer. Conjugation consists of transferring DNA fragments ranging from a few base pairs to large chromosomes or plasmids. The process of conjugation requires cell-to-cell contact, mating pair formation and transfer of plasmid DNA through a conjugative pilus. The genes for conjugative machinery are encoded by autonomously replicating plasmids or by integrative conjugative elements in the chromosome [39]. Conjugation rates in literature tend to vary a lot. One of the highest conjugation rates observed for plasmids is around ~10^−3^ transconjugants per recipient per hour in bacterial biofilms *in vitro*. According to the authors, these values are from one to three orders of magnitude greater than those reported by other studies [40], while the picture *in vivo* is slightly different. Conjugation rates have been examined using mice, yielding highly dissimilar results that vary from very low frequencies [41] to as high as ~7 × 10^−1^ transconjugants per donor cell, a value that was much higher than the rate found *in vitro* (~6 × 10^−8^) using the same strains [42]. These results suggest that the *in vitro* measured rates of genetic exchange are poor estimators of the *in vivo* frequencies.

In our previous experiments we found frequencies ranging from 10^−6^ to 10^−8^, which are comparable to most of conjugation experiments [24]. However, the experiments were carried out applying friction forces during one minute only. In the intestinal tract, discontinuous, but frequent friction forces of different intensities and duration are expected. Then, our *in vitro* results may underestimate the frequencies of this atypical HGT if it indeed occurs *in vivo*. Nevertheless, even very low frequencies of DNA transfer should be taken into consideration, as the possible consequences for human and animal biosafety could be of great importance. Furthermore, risk assessment should not be based solely on the observed frequencies, as they are not good predictors of long-term effects on bacterial communities [43].

## 3. Testing the Hypothesis

**Figure 2:**
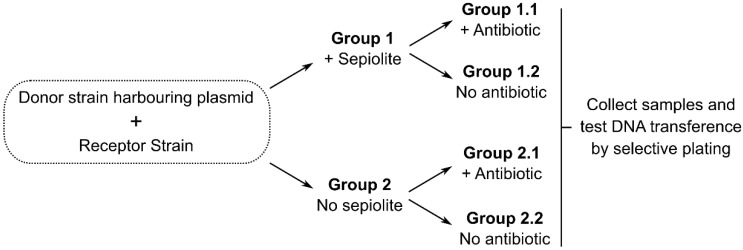
Ideal experimental model for testing the stated hypothesis. All germ-free animals in all groups should be given feed containing a safe bacterial strain harboring a resistance marker integrated in the chromosome or a known plasmid that cannot be transferred by conjugation (non-conjugative and non-mobilizable ones); this bacterium should carry an auxotrophy to allow for the counterselection of donors. A known recipient (for instance an *E. coli* strain different from the donor) could be used to easily verify transfer.

An ideal testing framework for our hypothesis may consist in a double blind, stratified experiment in which four groups of gnotobiotic animals are given feed containing or lacking sepiolite, an appropriate bacterial donor strain and antibiotics acting as selective force (Figure 2).

Faecal samples would then be collected to isolate bacteria in a medium designed to counterselect donor strains. Differences in the transfer of resistance genes would reflect the effect of the clay. Available reports on the efficiency of sepiolite in promoting bacterial transformation [24], the high sepiolite content of animal feed and the high bacterial content in faecal samples; it is expectable that even at low *in vivo* transfection efficiency and wide margins of error, this hypothesis might be accurately tested in a cost-effective way. However, a testing scenario would be strongly limited by the fact that experiments would require an S1 security level, and this is difficult to achieve with livestock animals. We should also admit that it is enormously difficult to detect rare HGT events and the resulting bacterial transformants within large heterogeneous microbial communities [44], which confer a poor resolution to this experimental approach. One possibility is the use of an animal model like in one study reporting vancomycin resistance gene transfer between enterococci of human origin in the gut of mice harbouring human microbiota [45]; although this would only provide an indirect evidence for livestock in case of positive results.

Epidemiological observations correlating the use of clay supplements with higher incidence of antibiotic resistance can be useful to initiate additional studies. In near future, the possibility of doing large comparative metagenomic studies in the microbiome of livestock may help to correlate the use of clay with altered frequencies of HGT. If our hypothesis is correct, “remodeled” bacteria are being transmitted from farms to communities. Clay materials in animal feed perchance significantly contribute to the spread of antibiotic resistance and virulence genes, increasing the problems of antibiotic ineffectiveness in the treatment of human and animal infections. This new mechanism of HGT may promote the acquisition of virulence factors and antibiotic resistance by gut pathogens with broad implications for humans and animals.

## 4. Conclusions

Here, we present a hypothesis raising the concern that under a livestock feeding regime, the confluence of factors such as antibiotic pressure, diversity and size of bacterial populations, the presence of potential pathogens, the particular physiology of digestive tract of cattle and clay present as a feed additive might favor clay-mediated horizontal gene transfer. The kind of transferred materials may consist of plasmids, and more importantly fragments of genomic DNA that may spread both antibiotic resistance and virulence factors between potential animal and human bacterial pathogens and transmit them to the community. We encourage the scientists from related disciplines to be aware about this possible mechanism and perform additional studies to see to what extent this HGT is operating.
